# Simultaneous Determination of Amphenicols and Metabolites in Animal-Derived Foods Using Ultrahigh-Performance Liquid Chromatography-Tandem Mass Spectrometry

**DOI:** 10.1155/2021/3613670

**Published:** 2021-11-03

**Authors:** Xinyi Wu, Xixi Shen, Xiangyue Cao, Rongrong Nie, Haonan Zhang, Changbo Tang, Wei Wang

**Affiliations:** Key Laboratory of Meat Processing and Quality Control, MOE, Key Laboratory of Meat Processing, MARA, Jiangsu Collaborative Innovation Center of Meat Production and Processing, Quality and Safety Control, College of Food Science and Technology, Nanjing Agricultural University, Nanjing 210095, China

## Abstract

Amphenicols are widely used to prevent and treat animal diseases. However, amphenicol residues accumulate in livestock and poultry and harm consumers. We hypothesized that one can combine solid-phase extraction (SPE) with ultrahigh-performance liquid chromatography-tandem mass spectrometry (UHPLC-MS/MS) to simultaneously determine amphenicols and metabolites in pork, beef, lamb, chicken, and their products and meet government regulations for maximum residue limits. We extracted crude samples with ethyl acetate and ammonia water (98:2, v/v), purified the samples with a CNW Si SPE column, defatted the samples with acetonitrile-saturated *n*-hexane, and then determined the resulting analytes by UHPLC-MS/MS. The limit of detection of the analytes in livestock and poultry meat was 0.03–1.50 *μ*g/kg, and the limit of quantification was 0.05–5.00 *μ*g/kg. Measured chloramphenicol, thiamphenicol, and florfenicol concentrations were linear over the range 0.50–50 *μ*g/kg; and the florfenicol amine concentration was linear over the range 5.00–200 *μ*g/kg (all with correlation coefficients >0.9990). The recovery of the spiked samples was between 72% and 120%. The intraday relative standard deviation (RSD) ranged from 1% to 9%, and the interday RSD ranged from 1% to 12%. Based on the above results, the current method is sensitive, accurate, and reproducible with the detection limits being well below the maximum residue limits as per Chinese standard GB 31650-2019, and thus, our research hypothesis could be confirmed.

## 1. Introduction

**Figure 1:**
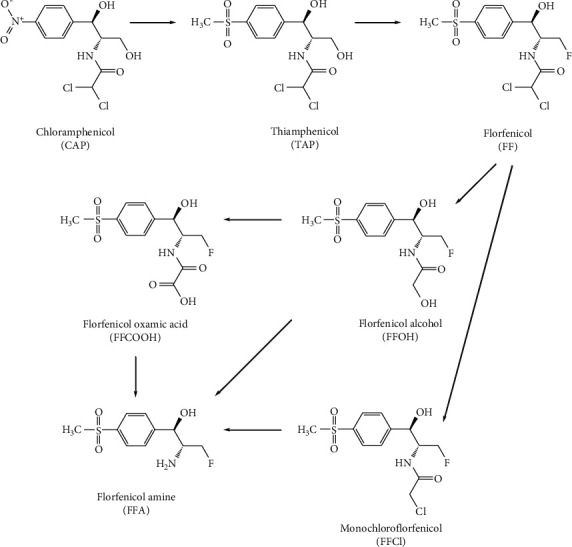
Chemical structures of amphenicols and metabolic route of FF.

China produces and consumes a large quantity of livestock and poultry meat; corresponding quality and safety problems harm consumer health and create barriers to the import and export trade [1]. Among these quality and safety problems, veterinary drug residues are especially pertinent. Chloramphenicol (CAP), thiamphenicol (TAP), and florfenicol (FF) are amphenicols, substances that are widely used in livestock and poultry breeding and production. These substances have broad-spectrum antibacterial properties, are low cost, and have high potency [2–4]. However, CAP is highly toxic to the human hematopoietic system and can cause side effects such as aplastic anemia [5, 6]. As a derivative of CAP, TAP replaces the *para*-nitro group of CAP with a methanesulfonyl group and is less toxic than CAP but can inhibit the production of red blood cells, white blood cells, and platelets [7, 8]. FF is synthesised from TAP by replacing the 3-hydroxy group with fluorine; it is an animal-specific amphenicol and has higher antibacterial activity than CAP and TAP but has embryo toxicity [9, 10]. FF is metabolized to florfenicol amine (FFA) via bioconversion pathways in treated animals (Figure 1) [11]. Therefore, to regulate the use of toxic amphenicols and reduce their prevalence in livestock and poultry products, the use of amphenicols is strictly restricted by laws and regulations. Currently, China, the United States, the European Union, Japan, and other countries have banned the use of CAP in animal feeds. As shown in Table 1, regulations in China, the United States (US), and the European Union (EU) have stipulated that the maximum residue limits (MRLs) of TAP in animal-derived foods such as the muscle, fat, liver, kidney, and milk are 50 *μ*g/kg; and the MRLs of FF (calculated as the sum of both FF and FFA) in the muscle, liver, kidney, skin, and fat are 100–3700 *μ*g/kg [12–17]. TAP is not recommended for animals from which eggs are produced for human consumption, and FF is not recommended for animals from which milk and eggs are produced for human consumption in China and the EU

Various approaches for the determination of amphenicols in animal-derived foods are available in the literature and apply detection systems such as biosensors [18–21], enzyme-linked immunosorbent assays [22, 23], high-performance liquid chromatography (HPLC) [24–26], liquid chromatography-tandem mass spectrometry (LC-MS/MS) [27–30], gas chromatography (GC) [31], and GC-MS/MS [32, 33]. Among them, LC-MS/MS is the main detection and positive confirmation method for amphenicols. Ultrahigh-performance liquid chromatography (UHPLC)-MS/MS is an LC-MS/MS technology that is more suitable than conventional LC-MS/MS for the analysis and detection of amphenicols because of a short analysis time, higher separation efficiency, and higher sensitivity. Currently, several analytical methods have been reported for the analysis of some of amphenicols and metabolites [28, 30, 34, 35]. In addition, these methods are usually developed for one or two matrices [7, 36, 37]. To our knowledge, there are few reports on simultaneously detecting the entire family of amphenicols and metabolites in animal-derived foods. In this study, we combined solid-phase extraction (SPE) with UHPLC-MS/MS in both negative and positive electrospray ionization (ESI) mode for simultaneous determination of CAP, TAP, FF, and FFA in pork, beef, lamb, chicken, and their products under the optimal experimental conditions. Our goal is to promote the development of international trade in animal-derived foods, provide references for quality and safety monitoring, and standardize supervision and testing methods of animal-derived foods.

## 2. Materials and Methods

### 2.1. Chemicals and Reagents

CAP, TAP, FF, FFA, and CAP-D5 standards were obtained from Dr. Ehrenstorfer GmbH (Augsburg, Germany). FFA-D3 standard was obtained from Toronto Research Chemicals (Toronto, ON, Canada). HPLC-grade acetonitrile (ACN), methanol, ethyl acetate, and *n*-hexane were supplied by Merck (Darmstadt, Germany). Reagent grade ammonium hydroxide, HPLC-grade acetone, and formic acid were supplied by Sinopharm Chemical Reagent Co. (Shanghai, China). Water was purified with an Arium Pro ultrapure water purification system (Sartorius, Göttingen, Germany).

### 2.2. Preparation of Standard Stock and Working Solutions

Standard stock solutions of CAP, TAP, FF, FFA, CAP-D5, and FFA-D3 were prepared at a concentration of 1000 *μ*g/mL by dissolving each analyte in methanol. All the aforementioned standard stock solutions were stored at −20°C for up to 6 mo.

Mixed standard working solutions of CAP, TAP, FF, and FFA at a concentration of 10 *μ*g/mL were prepared by diluting the standard stock solutions with methanol. An internal standard working solution at a concentration of 1.0 *μ*g/mL was prepared by diluting the internal standard stock solutions with methanol. The aforementioned standard working solutions were stored at −20°C for up to 3 mo.

### 2.3. UHPLC-MS/MS Instrumentation and Operating Conditions

A Thermo Scientific Vanquish ultrahigh-performance liquid chromatograph coupled to a Thermo Scientific TSQ Quantis mass spectrometer (Thermo Fisher Scientific, Waltham, MA, USA) was used. Separation was performed with a Waters Acquity UPLC high-strength silica (HSS) C18 column (50 mm × 2.1 mm, 1.8 *μ*m; Waters, Milford, MA, USA), with the front end connected to a Waters Acquity UPLC HSS C18 VanGuard precolumn (5 mm × 2.1 mm, 1.8 *μ*m; Waters, Milford, MA, USA). The column temperature was 40°C, and the sample injection volume was 2.0 *μ*L. Gradient elution was performed with water and ACN as the mobile phases, at a flow rate of 0.3 mL/min: 0–4 min, 4% (v/v) ACN increased to 96% (v/v); 4–6 min, 96% (v/v) ACN; and 6–10 min, 96% (v/v) ACN decreased to 4% (v/v).

The MS/MS was equipped with an ESI source operating in the positive ionization (PI) mode, negative ionization (NI) mode, and selected reaction monitoring mode. CAP, TAP, FF, and CAP-D5 were analyzed in the NI mode, whereas FFA and FFA-D3 were analyzed in the PI mode. The ESI source was operated with the following capillary voltage: 3.5 kV in PI mode and 2.5 kV in NI mode; sheath gas: 50 Arb; auxiliary gas: 10 Arb; ion transfer tube temperature: 325°C; and vaporizer temperature: 350°C.

### 2.4. Sample Preparation

Blank samples used in the validation procedure were obtained from the Supervision, Inspection, and Testing Center for Quality of Meat Products (Nanjing, China). Pork, beef, lamb, and chicken muscle were chopped and homogenized by an HM6300 intelligent homogenizer (Lab Precision Beijing Technology Co., LTD, Beijing, China) at 10,000 r/min, and aliquots of 5.0 g were weighed in a 50-mL polypropylene centrifuge tube. Samples were spiked with 10 *μ*L of the internal standard working solution, corresponding to a concentration of 2 *μ*g/kg of CAP-D5 and FFA-D3 in tissue. Then 15 mL of ethyl acetate with 2% (v/v) ammonia were added. The mixture was thoroughly homogenized using an IKA Vortex 3 (IKA, Staufen, Germany). After mixing, the homogenate was centrifuged at 10,621 g for 5 min at 4°C, and the supernatant was transferred into a 50-mL polypropylene tube. The extraction step was repeated by the addition of 15 mL of ethyl acetate with 2% (v/v) ammonia, shaken, and centrifuged again under the same conditions. The resulted supernatant was merged with the previous extract in the 50 mL polypropylene tube, and the extracts were evaporated to dryness at 40°C for 2.5 h under a nitrogen stream with an N-EVAP-11 nitrogen evaporator (Organomation, Berlin, MA, USA).

The residual solution was dissolved in acetone/*n*-hexane (1:9, v/v; 5 mL) and loaded onto a CNW Si SPE (200 mg) cartridge, which was preconditioned with water (5 mL) and acetone/*n*-hexane (1:9, v/v; 5 mL). The cartridge was eluted with acetone/*n*-hexane (6:4, v/v; 5 mL), and then the eluate was evaporated to dryness at 40°C for 10 min under a nitrogen stream. The residual solution was reconstituted by adding 1.0 mL of ACN-water (50:50; v/v) and then vortexed for 1 min. Next, 5 mL of hexane saturated with pure ACN was added to the tube, and the mixture was vortexed for 30 s. After centrifugation for 1 min at 10,621 g, the hexane layer was discarded. This defatting step was repeated twice. Finally, an aliquot of 1 mL of the bottom layer was poured through a 0.22 *μ*m filter into an autosampler vial, and then 2 *μ*L of the liquid in the aforementioned vial was analyzed using UHPLC-MS/MS.

## 3. Method Validation

### 3.1. Determination of Linearity, Limit of Detection, and Limit of Quantification of Amphenicols

A series concentration of matrix-matched standard solution was prepared by diluting the mixed standard working solutions with the blank matrix extract. Among them, CAP, TAP, and FF were set at 0.5, 1.0, 5.0, 10.0, and 50.0 *μ*g/kg within the range 0.5–50.0 *μ*g/kg, and FFA was set at 5.0, 10.0, 50.0, 100.0, and 200.0 *μ*g/kg within the range 5.0–200.0 *μ*g/kg (the concentrations of CAP-D5 and FFA-D3 were 10 *μ*g/L). The standard curves were constructed using the mass concentration of amphenicols (*μ*g/kg) as the abscissa (*x*) and the analyte/internal standard peak area ratio versus as the ordinate (*y*), and the squares of the correlation coefficients (*R*^2^) were calculated.

Negative livestock and poultry meat were added to the internal standard working solution and the mixed standard working solution and then detected with reference to the method described above. The limit of detection (LOD) was the minimum added concentration when the signal-to-noise ratio (S/N) ≥3, and the limit of quantification (LOQ) was the minimum added concentration when S/N ≥ 10 [38].

### 3.2. Recovery and Precision Test

Solutions were spiked into livestock and poultry meat as follows: standard working solutions of CAP, TAP, and FF at three concentration levels (1, 5, and 10 *μ*g/kg) and the standard working solution of FFA at three concentration levels (5, 10, and 50 *μ*g/kg). Pretreatment was performed in accordance with the method described above, and each concentration was set in six parallels. The recovery was calculated as follows:

(1) $$\begin{array}{c} \mathrm{recovery}\left(\%\right)=\frac{C_{E}}{C_{M}}\times 100\%, \end{array}$$

where *C*_*E*_ is the experimental concentration determined from the calibration curve and *C*_*M*_ is the spiked concentration. The precision was determined by calculating the relative standard deviation (RSD). Samples with three levels of spiked concentrations were measured in the same day and in six consecutive days, and then the intraday RSD and the interday RSD were calculated. The RSD was calculated as follows:

(2) $$\begin{array}{c} \mathrm{RSD}\left(\%\right)=\frac{\text{SD}}{C_{A}}\times 100\%, \end{array}$$

where SD is the standard deviation and *C*_*A*_ is the average of the experimental concentration determined from the calibration curve.

### 3.3. Application to Livestock Meat, Poultry Meat, and their Products

To validate our strategy, 28 real samples of commercial livestock meat, poultry meat, and their products from various locations in China were used to detect amphenicols. Amphenicols were extracted from the samples as previously described, and the extracts were analyzed with UHPLC-MS/MS.

### 3.4. Data Analysis

TraceFinder 4.1 software (Thermo Fisher Scientific, Waltham, MA, USA) was used for data acquisition and processing, and Origin 8.0 software (Microcal Software Inc., Northampton, MA, USA) was used for plotting.

## 4. Results and Discussion

### 4.1. Optimization of UHPLC-MS/MS Conditions

**Figure 2:**
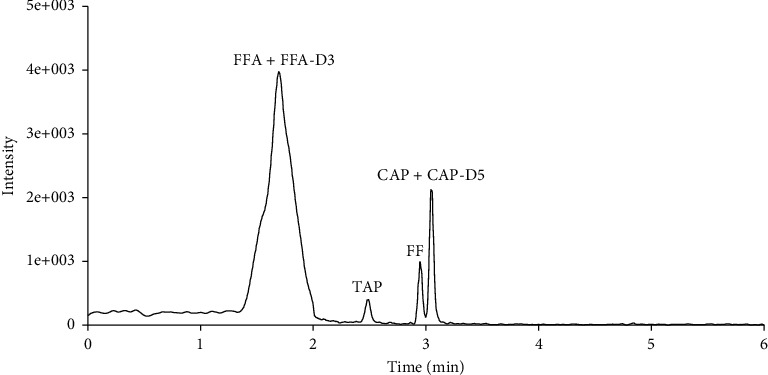
Chromatograms of CAP, TAP, FF, FFA, CAP-D5, and FFA-D3 at 10 *μ*g/L with an ACN-water mobile phase. CAP, chloramphenicol; FF, florfenicol; FFA, florfenicol amine; TAP, thiamphenicol.

The use of formic acid improved the response and peak shape corresponding to FFA but generated ion suppression of CAP, TAP, and FF—which we monitored in the NI mode [28]. Thus, to better separate amphenicols and metabolite residues, we used ACN-0.1 v/v% formic acid, ACN-0.2 v/v% formic acid, and ACN-0.3 v/v% formic acid as the mobile phase for UHPLC-MS/MS analyses in this experiment. The separation effect of the four target compounds was excellent when ACN-water was a mobile phase (Figure 2). The FFA peak became bimodal after adding formic acid to the mobile phase. Therefore, we selected ACN-water as a mobile phase to separate the four analytes.

We directly injected 1 *μ*g/mL of CAP, TAP, FF, FFA, CAP-D5, and FFA-D into the mass spectrometer at a flow rate of 20 *μ*L/min, and the instrument scanned in Full Scan Q1 simultaneously in ESI (+) and ESI (−) to determine the precursor ion of each compound. Then the instrument scanned in SIM Q3. For each compound, we optimized two pairs of characteristic ion pairs with the highest response as qualitative and quantitative ion pairs. We also optimized the declustering voltage and collision energy of each ion pair. In doing so, we obtained corresponding mass spectrometry parameters (Table 2).

### 4.2. Optimization of Sample Preparation

The extraction solvent was ACN, which showed satisfactory recoveries for CAP. However, when the other analytes were introduced in the method, it was necessary to choose the extraction solvent to provide better recoveries rates for all analytes, not just for CAP. The solvent of election was ethyl acetate, which can promote the extraction of all analytes. Moreover, the use of ammonium hydroxide as an additive can promote an alkaline environment, which is favorable for the extraction of FFA. Taking into account the extraction efficiency of all the amphenicols and metabolites, it was necessary to choose an extraction solvent that was able to provide better recoveries for all the analytes. Therefore, we chose the following extraction methods for comparison:

- Method A: ethyl acetate: ammonium hydroxide (98:2, v/v), extract twice
- Method B: ACN: ammonium hydroxide (98:2, v/v), extract twice
- Method C: ACN, extract twice
- Method D: ACN: ethyl acetate: ammonium hydroxide (49:49:2, v/v/v), extract twice
- Method E: ethyl acetate: ammonium hydroxide (98:2, v/v), extracted once; and ACN: ammonium hydroxide (98:2, v/v), extracted once

After the extraction by method A, the recovery of amphenicols and metabolites in the four livestock and poultry meat products was all in the range 60% to 120% (Table 3). Therefore, we chose method A as the optimal extraction method.

Because livestock and poultry meat contain a large quantity of fat and protein and are complex matrices, the samples require further purification after extraction. Therefore, we compared the purification effects of the following columns: HyperSep Retain PEP (60 mg/3 mL; Thermo Fisher Scientific, Waltham, MA, USA), Oasis HLB (200 mg/6 mL; Waters, Milford, MA, USA), and CNW Si SPE (60 mg/3 mL; ANPEL Experimental Technology Co., Shanghai, China). After purification with the CNW Si SPE column, the recovery of amphenicols and metabolites in the four livestock and poultry meat products was in the range from 60% to 120% (Table 4). Therefore, we chose the CNW Si SPE column as the optimal purification method.

### 4.3. Linearity of Standard Curves, LOD, and LOQ for Amphenicols and Metabolites

We used CAP-D5 as the internal standard for CAP, TAP; and FF; and FFA-D3 as the internal standard for FFA. In four types of livestock and poultry meat, the method was linear over the range of 0.5–50.0 *μ*g/kg for CAP, TAP, and FF; and of 5.0–200.0 *μ*g/kg for FFA. The *R*^2^ was greater than >0.999. The LOD and LOQ of the four tested compounds ranged from 0.03–1.50 *μ*g/kg and 0.11–5.0 *μ*g/kg in four types of livestock and poultry meat (Table 5). Compared with other literature reports [27,28,38], this method is fast and has high sensitivity and meets the requirements of the National Food Safety Standard on Maximum Residue Limits for Veterinary Drugs in Foods (GB 31650–2019) in China.

### 4.4. Recovery and Precision of Amphenicols and Metabolites

To verify the recovery and precision of this method, we spiked blank samples at three concentrations within the ranges 1.0–10.0 *μ*g/kg for CAP, TAP, and FF and 5.0–50.0 *μ*g/kg for FFA (*n* = 6 at each level).  Table 6 summarizes the results of the recovery and precision experiments. The average recovery of the four analytes in livestock and poultry meat samples was 71.77% to 120.30%, with an intraday RSD <9.22% and interday RSD <12.30%. Thus, our method has high recovery and good precision and can achieve simultaneous detection of amphenicols and metabolites in livestock and poultry meat.

### 4.5. Real Sample Analyses

We selected 28 commercial livestock meat, poultry meat, and their products to verify practical applications of our method in real food matrices with possible interferences. We used one positive meat sample as the quality control sample. We detected 1.38 *μ*g/kg of the banned veterinary drug CAP in fresh chicken, 0.35 *μ*g/kg of FF in pork sausage, and 0.54 *μ*g/kg of FF in fried pork jerky (Qingdao, Shandong), yet none of the samples exceeded the MRL of 300 *μ*g/kg for porcine muscle (GB 31650-2019; Table 7).

## 5. Conclusions

Amphenicols are widely used in livestock and poultry breeding and production, but illegal or noncompliant use may occur. This causes amphenicol residues to accumulate in the body of livestock and poultry animals. Such accumulation is a threat to livestock and poultry food safety, as well as human health. Therefore, we developed a detection method for amphenicols and metabolites in livestock meat, poultry meat, and their products. By optimizing MS, LC, and sample pretreatment conditions, we established a UHPLC-MS/MS detection method that can simultaneously detect amphenicols and metabolites in livestock meat, poultry meat, and their products. The peak time of the analytes was within 5 min; the LOD was 0.03–1.50 *μ*g/kg; the LOQ was 0.05–5.00 *μ*g/kg; the recovery of spiked samples at low, medium, and high concentrations was between 71.77% and 120.30%; the intraday RSD was <9.22%, and the interday RSD was <12.30%. Thus, this method has a short analysis time, a high sensitivity, and a good repeatability. We have thus provided scientific support and technical assurance for reliable detection of amphenicols and metabolites in animal-derived foods for food safety monitoring.

## Figures and Tables

**Table 1:** Comparison of the MRLs for amphenicols in China, the US, and the EU.

Analyte	Species	Target tissue	MRL (*μ*g/kg)		
			China	US	EU
TAP	All species	All tissues	50		50

FF (sum of FF and FFA)	Porcine	Muscle	300	200	300
		Skin and fat	500		500
		Liver	2000	2500	2000
		Kidney	500		500
	Bovine	Muscle	200	300	200
		Liver	3000	3700	3000
		Kidney	300		300
	Ovine caprine	Muscle	200		200
		Liver	3000		3000
		Kidney	300		300
	Poultry	Muscle	100		100
		Skin and fat	200		200
		Liver	2500		2500
		Kidney	750		750

**Table 2:** Mass spectrometry conditions and range of relative intensities for each compound.

Analyte	Polarity	Precursor ion (*m*/*z*)	Product ion (*m*/*z*)	Declustering voltage (V)	Collision voltage (V)
CAP	−	321	257	113	−10
			152^*∗*^	113	−12

CAP-D5	−	326	157	113	−16

TAP	−	354	290^*∗*^	290	−12
			185	164	−10

FF	−	356	185^*∗*^	141	−20
			119	141	−38

FFA	+	248	230^*∗*^	100	10
			130	100	27

FFA-D3	+	251	233	85	10

^*∗*^Quantification ion. CAP, chloramphenicol; FF, florfenicol; FFA, florfenicol amine; TAP, thiamphenicol.

**Table 3:** Recoveries of amphenicols and metabolites under various extraction methods.

Extraction method	Matrix	Number of amphenicols and metabolites		
		Recovery <60%	Recovery 60% to 120%	Recovery >120%
A	Pork	0	4	0
	Beef	0	4	0
	Lamb	0	4	0
	Chicken	0	4	0

B	Pork	1	1	2
	Beef	1	3	0
	Lamb	1	1	2
	Chicken	3	1	0

C	Pork	1	1	2
	Beef	1	2	1
	Lamb	1	1	2
	Chicken	1	3	0

D	Pork	1	1	2
	Beef	1	2	1
	Lamb	0	2	2
	Chicken	0	4	0

E	Pork	1	2	1
	Beef	1	1	2
	Lamb	0	2	2
	Chicken	2	2	0

**Table 4:** Recoveries of amphenicols and metabolites under various purification methods.

Purification method	Matrix	Number of amphenicols and metabolites		
		Recovery <60%	Recovery 60% to 120%	Recovery >120%
HyperSep Retain PEP	Pork	1	1	2
	Beef	0	4	0
	Lamb	1	2	1
	Chicken	0	2	2

Oasis HLB SPE	Pork	2	0	2
	Beef	0	3	1
	Lamb	1	2	1
	Chicken	1	1	2

CNW Si SPE	Pork	0	4	0
	Beef	0	4	0
	Lamb	0	4	0
	Chicken	0	4	0

**Table 5:** Linearity of standard curve, *R*^2^, LOD, and LOQ of amphenicols and metabolites in livestock and poultry meat.

Matrix	Analyte	Linearity (*μ*g/kg)	Linear equation	*R* ^2^	LOD (*μ*g/kg)	LOQ (*μ*g/kg)
Pork	CAP	0.50–50.0	*y* = 0.05933*x* + 0.02204	0.9996	0.11	0.36
	TAP	0.50–50.0	*y* = 0.01864*x* + 0.02831	0.9992	0.12	0.37
	FF	0.50–50.0	*y* = 0.1219*x* + 0.05289	0.9998	0.03	0.11
	FFA	5.00–200.0	*y* = 0.07707*x* − 0.00162	0.9991	0.60	2.00

Beef	CAP	0.5–50.0	*y* = 0.06262*x* − 0.01198	0.9995	0.05	0.18
	TAP	0.5–50.0	*y* = 0.01741*x* + 0.006421	0.9991	0.06	0.20
	FF	0.5–50.0	*y* = 0.1233*x* − 0.09559	0.9992	0.04	0.13
	FFA	5.0–200.0	*y* = 0.06948*x* − 0.9006	0.9998	1.50	5.00

Lamb	CAP	0.5–50.0	*y* = 0.05427*x* + 0.07979	0.9995	0.06	0.21
	TAP	0.5–50.0	*y* = 0.02154*x* + 0.02831	0.9992	0.06	0.21
	FF	0.5–50.0	*y* = 0.1553*x* + 0.2424	0.9998	0.04	0.12
	FFA	5.0–200.0	*y* = 0.05435*x* − 0.1845	0.9992	0.50	5.00

Chicken	CAP	0.5–50.0	*y* = 0.08144*x* − 0.1681	0.9991	0.03	0.05
	TAP	0.5–50.0	*y* = 0.03328*x* − 0.04784	0.9992	0.03	0.11
	FF	0.5–50.0	*y* = 0.1599*x* − 0.2144	0.9991	0.03	0.11
	FFA	5.0–200.0	*y* = 0.05276*x* − 0.1651	0.9991	1.00	3.00

CAP, chloramphenicol; FF, florfenicol; FFA, florfenicol amine; LOD, limit of detection; LOQ, limit of quantification; TAP, thiamphenicol.

**Table 6:** Recovery and precision of amphenicols and metabolites (*n* = 6).

Matrix	Analyte	Added level (*μ*g/kg)	Recovery (%)	Intraday RSD (%)	Interday RSD (%)
Pork	CAP	1	98.93	4.90	6.38
		5	115.81	2.16	3.71
		10	113.16	5.00	5.29
	TAP	1	95.80	8.30	9.11
		5	112.92	7.44	7.82
		10	106.31	6.93	7.92
	FF	1	83.81	5.62	5.68
		5	93.02	2.82	4.59
		10	95.63	1.83	4.12
	FFA	5	97.07	2.51	6.24
		10	89.22	6.23	10.42
		50	89.61	7.93	11.94

Beef	CAP	1	113.00	7.44	7.31
		5	99.73	4.41	8.24
		10	93.30	2.50	10.35
	TAP	1	87.91	5.53	9.47
		5	93.99	5.10	11.90
		10	80.10	3.02	7.10
	FF	1	80.13	6.08	8.22
		5	89.50	4.90	4.93
		10	96.81	2.12	2.41
	FFA	5	86.43	9.22	9.60
		10	80.29	7.18	7.22
		50	100.22	3.90	10.8

Lamb	CAP	1	97.20	1.83	2.30
		5	110.91	2.61	6.00
		10	120.30	3.88	7.43
	TAP	1	71.77	6.15	6.20
		5	105.44	4.50	4.86
		10	99.02	1.93	2.83
	FF	1	81.21	6.27	12.30
		5	109.00	1.21	1.37
		10	99.03	1.90	4.19
	FFA	5	101.14	4.58	11.00
		10	103.91	5.24	8.25
		50	92.02	3.72	8.33

Chicken	CAP	1	101.27	4.71	8.34
		5	101.05	1.63	3.60
		10	97.03	5.74	5.42
	TAP	1	84.00	6.88	11.70
		5	105.23	6.30	8.70
		10	103.54	6.91	11.02
	FF	1	101.10	3.90	5.22
		5	85.44	7.16	9.70
		10	82.82	4.28	4.21
	FFA	5	108.71	6.30	5.54
		10	101.63	6.62	11.30
		50	89.90	7.30	7.64

CAP, chloramphenicol; FF, florfenicol; FFA, florfenicol amine; RSD, relative standard deviation; TAP, thiamphenicol.

**Table 7:** Test results of commercial livestock meat, poultry meat, and their products.

Sample name	Place of origin (China)	CAP	TAP	FF	FFA
Fresh pork (positive control)	Nanjing, Jiangsu	+	+	+	+
Fresh pork	Nanjing, Jiangsu				
Pork sausage	Linyi, Shandong			+	
Pork ham	Nanjing, Jiangsu				
Fried pork jerky	Zibo, Shandong				
Fried pork jerky	Qingdao, Shandong			+	
Roasted pork jerky	Zhangzhou, Fujian				
Roasted pork sausage	Yantai, Shandong				
Fresh beef	Nanjing, Jiangsu				
Spiced beef	Pingxiang, Jiangxi				
Beef sausage	Linyi, Shandong				
Fried beef jerky	Bazhong, Sichuan				
Fried beef jerky	Zibo, Shandong				
Charcoal grilled beef jerky	Tongliao, Nei Mongol				
Charcoal grilled beef jerky	Chifeng, Nei Mongol				
Fresh lamb	Nanjing, Jiangsu				
Boiled lamb	Suzhou, Jiangsu				
Spiced lamb	Hulunbuir, Nei Mongol				
Fried lamb jerky	Tongliao, Nei Mongol				
Fried lamb jerky	Bayannaoer, Nei Mongol				
Roast leg of lamb	Hulunbuir, Nei Mongol				
Roast leg of lamb	Xilin Gol League, Nei Mongol				
Fresh chicken	Nanjing, Jiangsu	+			
Instant chicken breast	Pingxiang, Jiangxi				
Chicken sausage	Linyi, Shandong				
Crispy chicken	Nanjing, Jiangsu				
Fried chicken	Nanjing, Jiangsu				
Roast chicken	Yantai, Shandong				
Beggar's chicken	Hangzhou, Zhejiang				

+, detected; no entry indicates not detected. CAP, chloramphenicol; FF, florfenicol; FFA, florfenicol amine; TAP, thiamphenicol.

## Data Availability

The original data used to support the findings of this study can be obtained from the corresponding author upon request.
